# Integrating Parasite Eradication with Family Planning: The Colonial Legacy in Post-War Medical Cooperation in East Asia

**DOI:** 10.1093/shm/hkaa005

**Published:** 2020-06-23

**Authors:** Aya Homei, John P DiMoia

**Affiliations:** 1 School of Arts, Languages and Cultures, University of Manchester, Manchester, UK; 2 Department of Korean History, College of Humanities, Seoul National University, South Korea. E-mail: jdimoia@snu.ac.kr; 3 Lecturer in Japanese Studies at the University of Manchester; 4 Associate Professor of Korean History at Seoul National University

**Keywords:** Japan, South Korea, development aid, family planning, parasite control

## Abstract

This article depicts how anti-parasite and family planning campaigns developed in Japan and Korea independently after the Second World War, as specifically domestic public health initiatives that directly contributed to the post-war reconstruction (Japan) and nation-building (South Korea) exercises, and examines how they were later incorporated into development aid projects from the 1960s. By juxtaposing domestic histories of Japan as a former coloniser, and South Korea as its former colony, the article explores colonial legacies in post-war medical cooperation in East Asia. Furthermore, by clarifying how Japanese and South Korean development aid projects both grew from the *links* that existed in their respective domestic histories, the article aims to highlight complexities engrained in the history and to shed new light on a historiography that often locates the origins of development aid in colonial history.

The practice of colonial rule under Japanese empire encompassed large parts of Northeast (Taiwan, Korea and Northeast China/Manchuria) and Southeast Asia and is often characterised in terms of its period of political rule. However, its legacy extends beyond these temporal (late nineteenth century to mid-twentieth century) and geographical boundaries, including a diverse set of bureaucratic structures and forms of knowledge production. The 1945 dividing line does not represent a neat periodisation; and in fact, the break-up of Japanese empire allowed for new opportunities as informal networks previously maintained under empire started to assume new collaborative forms within ‘Free World’ Asia. This essay examines the medical collaboration undertaken between Japanese and Korean elites beginning in the late 1960s, a joint project comprising anti-parasite public health activities, along with elements of family planning (FP), a conjoined set of interests culminating in the formation of APCO (Asian Parasite Control Organization) in 1974. Although APCO bore the approval of official recognition, it emerged from several distinct clusters of private interests, with Japanese and Korean actors taking advantage of these emerging opportunities to further their distinct national and regional projects.

If it is critical to recognise these informal origins, a second key point for APCO concerns its supporting narrative of Asian fraternal relations, a powerful message in the post-war period. Despite the conspicuous overlap with the project of pre-war Japan, Japanese participants successfully mobilised their ideas and practice as the benefit of working with a ‘new’ Japan, one possessing developmental expertise and eager to provide international assistance. If these actors sought to reinvent themselves, a similar claim can be made for the Korean actors, many of whom had received their higher education under colonial rule. In post-colonial South Korea, these emerging elites could elide their controversial past—one frequently characterised as that of a ‘collaborator’, or *Ch’inilp’a* (‘pro-Japanese’)—and provide new solutions in both the domestic and regional spheres. The second of these became important when South Korea engaged with the Vietnam War (1964–73), providing civic and medical assistance, along with a military presence. By 1974, therefore, APCO’s formation appeared somewhat distant from its origins in Japanese empire and the medical ambitions of the 1930s and 1940s, emerging instead from these sets of informal networks and a carefully crafted message of pan-Asian knowledge exchange.

Reflecting the growing interest in ‘global history’ in the History of Science, Technology and Medicine (HSTM), works engaging with questions of how colonial legacies manifested in science, technology and medicine for the postcolonial world abound. Works of the kind that are relevant to our article include, for instance, Uma Kothari’s, which points out how ‘development studies’ as a field of expertise in social science established after the Second World War directly drew on colonial expertise in India under British colonial rule.^1^ Karl Ittmann has made a similar observation in his examination of the formation of demography in Britain before and after the war.^2^ Finally, recent literature includes the work of Liat Kozma, who has clarified how colonial legacies affected postcolonial and international medical theories of public health and hygiene by examining the so-called *bejel*, a form of syphilis endemic in the Arab world.^3^ These works almost unanimously resist the conventional periodisation predicated on the notion of colonialism solely as a political project of fixed duration.

To turn to the scholarship for East Asian History of Science, Technology and Medicine (EAHSTM), the expanding body of literature examines how the experience of Japanese colonial rule shaped the reconfiguration of science, technology and medicine in East Asia in the post-war period that was informed by a developmentalist agenda and emerging national development aid policies.^4^ With regard to colonial legacies, these works largely agree with the argument presented in the aforementioned works, but there are two interlinked perspectives characteristic to this specific body of literature. The first is a sustained gaze onto the nation-state. Given that nation-states played a pivotal role in shaping EAHSTM towards the common goal of nation-building in the region, this perspective has been inevitable. The second is the focus on the intersection between domestic and international politics. The works have confirmed how shifting regional geopolitics (from one buttressed by Japanese and western colonialisms to the post-war and Cold War power constellation) and international/transnational agenda were at the heart of what at a glance seemed very much state-focused success stories of post-war development projects in which STM played a critical role. However, their primary focus on one national context or form of STM mobilised by a bilateral agreement has thus far precluded us from drawing a comprehensive map of regional-cum-transnational constellations that constituted complex layers of interactions in which STM unfolded in development projects.^5^

Against this backdrop, this article depicts how anti-parasite and FP campaigns developed in Japan and Korea first as specifically domestic public health initiatives contributing to the post-war reconstruction (Japan) and nation-building (South Korea) exercises which then were incorporated into development aid projects from *c.* 1960s.^6^ The specific focus on Japan and Korea is advantageous on a couple of points to understand colonial legacies in post-war HSM in East Asia. First, by showing how similar domestic health campaigns (anti-parasite and FP) developed in two national contexts after the Second World War in the case of Japan and the Korean War in South Korea, we confirm the point advanced by DiMoia and Suzuki & Aldous that highlights how pivotal mass health projects were for the purpose of post-war nation-(re)building.^7^ Yet, second, different political statuses of each country during Japan’s colonial rule (Japan as a ruler and Koreas the ‘colonised’) shaped the specific ways in which colonialism manifested as rhetoric and legacy in post-war domestic health campaigns, as well as overseas development aid initiatives.^8^ By describing the differences, this article contributes to the growing body of new work, such as the one by Barak Kushner and Sherzod Muminov, which highlights how unevenness characterised the ways in which the demise of the Japanese empire and the emerging Cold War reshaped cultural, social and political life across East Asia.^9^ Furthermore, by showing the *links* between Japan and South Korea, as well as parallel trajectories in the ways in which colonial legacies and transnational agenda mobilised STM for overseas development aid, we aim to stress how complexities characterised the regional development aid campaigns that could not be accounted for by the imperial and sub-imperial distinctions.^10^

With these points in mind, we will examine three specific case studies, each of which had different trajectories and drew from distinctive narratives and infrastructural trajectories. The first is the Japan–South Korea medical cooperation in parasite control in the 1960s.^11^ The second is South Korean medical cooperation in Vietnam unfolding in parallel with the Japan–South Korea medical cooperation. Finally, the third pertains to the formation of the allegedly ‘pan-Asian’ anti-parasite/FP campaign launched by the Japanese health activist Kunii Chōjirō and developed by the network of Japanese, South Korean and Taiwanese parasitologists, FP activists and technocrats in the early 1970s.

## Non-governmental Health Campaigns in the Context of National Reconstruction: Kunii Chōjirō in Parasite Control and FP Initiatives, 1948–1960

Since the Meiji period, the idea of individuals’ health as essential to national power exhorted the Japanese state to discipline the bodies of its subjects under the name of public health.^12^ The trend continued despite shifting political environments. In the late 1940s, under the Allied Occupation (1945–52), the Ministry of Health and Welfare of the Japanese government and the Public Health & Welfare Section (PH&W) of the Supreme Commander for the Allied Powers General Headquarters (SCAP-GHQ) regarded public health reform as a priority area. The reasons for this approach varied, but the consensus between the occupiers and the occupied that public health formed a basis for the reconstruction of war-torn Japan was a major driving force. From the Japanese point of view, public health reform represented an additional way of disciplining Japanese bodies and health behaviours that would be particularly pertinent to post-war Japan.^13^ In turn, according to Aldous and Suzuki, American occupiers considered the modernisation of the Japanese public health system an effective tool to democratise Japan that would ultimately transform the nation, one of the Occupation’s central missions.^14^ The converging interests between the Japanese and the Occupied authorities towards the reconstruction of the Japanese nation catalysed post-war public health reform.

Campaigns to eradicate intestinal parasite infections emerged as a topic of discussion under these circumstances.^15^ Within the officialdom, General Crawford F. Sams, chief of the PH&W, endorsed public health initiatives aimed to change the sewage system and the custom of using human excrement as fertiliser, both of which were attributable to the proliferation of soil-transmitted parasites. However, immediately after the war, government officials were noncommittal to parasite infections. At the time, health officials were compelled to tackle other, more deadly life-threatening infectious diseases. Under the circumstance, such benign infections as the ones caused by intestinal parasites remained a low priority.^16^

However, the aforementioned circumstances granted an opportunity for Kunii Chōjirō (1916–96) to develop parasite eradication as a non-governmental health campaign. Kunii entered into health activism by accident. Kunii originally studied French literature at university and during the wartime, served the army as a low-rank soldier. After the war, with his fellow countryman Sugiyama Jirō, he tried to implement a Chinese-style industrial cooperative scheme in Japan.^17^ The campaign was not going well when, in October 1948, he fell ill. He eventually found out he had been suffering from hookworm infestation and recovered within a matter of a week, thanks to a drug. Afterwards, Kunii ditched the cooperative movement and moved on to parasite eradication.^18^

The first thing Kunii did when he jumped into the new campaign was to establish a network. Through his existing contacts, Kunii gained access to Koizumi Makoto, an eminent parasitologist at Keio University. Based on the new contact, he then organised specific projects. In January 1949, Kunii launched a parasite screening and eradication scheme for school children in the Tokyo area with his fellow activists Kubo Yoshio and Inami Kazuyoshi.^19^ Meanwhile, Kunii proposed to Koizumi to form an organisation dedicated to parasite control where activists and specialists could collaborate. On 24 June 1949, the Tokyo Association for the Prevention of Parasite Infection was established, with Koizumi as a chairperson of the board of trustees. Throughout the first half of the 1950s, the membership of the Tokyo Association increased annually. Along with it, the campaign’s reach expanded across the country. Eventually, in 1955, Kunii helped to establish the Japanese Association for the Prevention of Parasite Infection (JAPPI) as a nationwide non-governmental organisation aimed to eradicate parasite diseases by implementing a screening practice at schools and by teaching screening techniques among the community public health technicians. In the 1950s, the campaign was booming.

The success of Kunii’s parasite control campaign was certainly attributable to the aforementioned opportunity laid out by the officialdom, but this was not the whole story. The awareness of the cost issues held a key to the success, too. First, the specific campaign could be run at a low cost by capitalising on the local public health infrastructure and school system that had been radically reformed under the Occupation.^20^ Secondly, the cost could be kept even lower, because parasite screening and prevention did not (theoretically) require participation of expensive medics, though their cooperation would be desirable. Thirdly, infections of intestinal parasites could be easily cured with a relatively inexpensive drug, and the dramatic effect of the initiative could work in favour of the campaign. It was these calculations that informed Kunii’s decision to opt for parasite control in his health campaign.

Despite the campaign’s success, from around 1953, Kunii began to seek for another cause and ultimately landed on FP. The switch to the new cause, as accounted by Kunii, was based solely upon his individual decision. However, similar to the parasite eradication campaign, the historical context also played a pivotal role in steering him towards FP. Japan immediately after the war witnessed a quick population growth, which was attributed to the post-war ‘baby boom’ and the repatriation of soldiers and civilians living in Japan’s former colonies. Policy advisors and policymakers, both in the Japanese government and SCAP-GHQ, saw post-war ‘overpopulation’ (*kajō jinkō*) as a threat to national reconstruction, either by catalysing or exacerbating the socio-economic problems confronting Japan, such as food shortage, land erosion and unemployment. In this context, on 26 October 1951, the Japanese Cabinet decided to adopt FP as a national policy. In 1952, the Eugenic Protection Law, originally issued in 1948, was amended to facilitate the new policy. The amendment stipulated that FP counselling should be implemented in the community-level eugenic marriage office. It also created a new category of health professionals, ‘FP field instructors’, trained and certified on the local government level to give public seminars on birth control, advise married couples on specific contraceptives and sell certain contraceptives at the wholesale price.^21^

In contrast to the civilian parasite eradication campaign, birth control activism had a longer history dating back to the 1920s.^22^ However, until after the war, the government held an ambiguous, if not outright hostile, attitude to the movement precisely because of its association with socialism and, during the war, because of pro-natalism. After the war, due to the drastic political change, birth control activism resumed. In the late 1940s and 1950s, aided by the aforementioned political discourse upholding birth control, the movement flourished. However, even in the early 1950s, there was no one united grassroots birth control organisation that the government could liaise with. Nevertheless, discussions were taking place among the noted individuals within the movement to establish a nationwide FP organisation that would act as a Japanese partner of the International Planned Parenthood Federation (IPPF). In April 1954, in part to commemorate Sanger’s visit to Japan, the Family Planning Federation of Japan (Nihon Kazoku Keikaku Renmei, thereafter FPFJ) was established.

Kunii entered the field at this specific moment. In the early 1950s, Kunii began to wonder about the financial viability of the anti-parasite activism.^23^ Success in the campaign would signify healthy people free from parasite infection, which could easily lead to the demise of the campaign. Just at the moment, in 1953, Kunii met Hinoue Sadao, a Ministry of Health and Welfare bureaucrat who had recently been appointed to take charge of the FP matter. Hinoue told Kunii that the government was so far unable to mobilise the birth control policy and explained that it was due to the lack of public involvement. He then suggested that Kunii organise non-governmental campaigns to aid the government initiative. Kunii jumped on the bandwagon; with Hinoue and his boss Ozawa Tatsuo, he planned to organise a private FP organisation. On 20 April 1954, with the publication of *Family Planning*, Kunii launched the Japan Family Planning Popularization Association (JFPPA).^24^

Among the JFPPA’s activities, publication of *Family Planning* formed a critical part, but it was the sales of condoms that supported the organisation’s core service and finance. The condom business started in the summer of 1954, when Kunii had an opportunity to visit and interview the condom manufacturer Okamoto Rubber as part of the delegates. After the visit, Kunii managed to negotiate with Okamoto Rubber to buy condom packs at the reduced price of 30 yen. From 1955, the JFPPA sold them to the prefectural hygiene bureaus and corporations at 40–50 yen, at a time when the retail price was set around 100 yen.^25^ Selling condoms was not entirely devoid of criticism.^26^ However, Kunii was cognizant that the JFPPA, as non-governmental organisation with limited funding, needed a source of support. Out of many commercial options, Kunii viewed that the sales of condoms were appropriate for the JFPPA.

After the launch of the JFPPA, Kunii quickly established himself to be one of the leading figures in the FP activism circle. The great opportunity came immediately, when Kunii and Katagiri Tameise, his fellow activist at the JFPPA, volunteered to take charge of publicity for the Fifth International Conference on Planned Parenthood held in Tokyo in October 1955.^27^ The Congress organisers valued their work, which raised the reputation of Kunii and his JFPPA. Thereafter, Kunii assumed leadership positions in various private-sector FP organisations, which became a basis for his transnational activism from the late 1960s onwards.

Kunii’s activism indicates the important aspect of national reconstruction in early post-war Japan realised via health reforms that it was hardly a predetermined, highly systematic or top-down process led by the national government. Instead, it heavily relied on the grassroots initiatives born out of rather frivolous and chaotic circumstances after the war and on the interaction between the government and non-government agencies. Kunii rose to prominence in this specific context. Over the 1960s, as the Japanese government developed medical cooperation programmes and actively promoted parasite eradication and FP in Asia as development aids, Kunii applied the experience and networks he accumulated in the 1950s and helped to establish the non-governmental Japanese Organization for International Cooperation in Family Planning (JOICFP) in 1969. Over the 1970s, JOICFP, as the only non-government organisation specialising in international cooperation in FP in Japan, developed FP initiatives by collaborating with the government’s development aid and international cooperation agencies, Overseas Technical Cooperation Agency (OTCA, est. June 1962) and its successor Japan International Cooperation Agency (JICA, est. August 1974).

Meanwhile, campaigns to eradicate intestinal parasite infections and to spread FP practices took place in South Korea. Similar to post-war Japan, these health initiatives were defined in relation to the nascent post-war nation-state and mediated through the cooperation of political figures, doctors and health activists.

## The Korean Medical Community in Transition

The background to the development of a national anti-parasite campaign (1969 to early 1990s) in South Korea holds a lengthy history, one linking it with the legacy of Japanese imperial parasitology (1910–45), the domestic problem of rural public health and the subsequent project of South Korean nation-building.^28^ The majority of Korean specialists in parasitology and, later, tropical medicine received Japanese training, if they were fortunate, to the university level. After 1945, many of these same individuals, ready to assume leading positions, found themselves strategically placed to take advantage of new opportunities to gain access to emerging international networks, especially following the Korean War (1950–53), with scholarships becoming available in the USA.^29^ In practice, this pattern of movement suggests a hybrid set of models and professional vocabulary, one not necessarily indebted to any single approach, although clearly the Japanese influence remained strong for some time. By the early 1960s, South Korea was ready to address its parasite problem, here referring specifically to the gastrointestinal tract and the effects of the post-war Korean diet.^30^

As previously noted, Japan’s renewed interest in parasites dates to the American occupation (1945–52), with the post-war bringing an encounter with poverty and circumstances of deprivation. For South Korea, there was a comparable investment in the problem, as the combined force of the USAMGIK (United States Army Military Government in Korea) occupation (1945–48), distinct from that of GHQ in Japan, was compounded by the Korean War, bringing with it devastation. Moreover, academic parasitology, associated with the university community, and the Japanese role in policing its colonies remained a strong influence, given the small, but vibrant, group of Koreans trained over the course of the previous two decades.^31^ The youngest generation among these individuals possessed the expertise to address a growing national problem. A lack of chemical fertiliser after 1948, coupled with the war, meant that an agrarian nation was dependent almost entirely upon ‘night soil’ to replenish nitrates to its fields.^32^ Koreans consumed and recirculated a wide range of parasites every time they ate, a growing concern both in terms of health and the nation’s self-image.^33^

The first decade following the close of combat witnessed reconstruction efforts in public health, although much of this activity was devoted to training and restoring basic infrastructure. The colonial period had seen limited chances for Koreans, especially for higher education. A number of newly established exchange relationships, especially the one between Seoul National University and the University of Minnesota (1954–62) took form, with a range of institutions receiving sources of external funding.^34^ European medical authorities also sought to provide assistance, with the Scandinavian Project (1958–68) taking shape at Seoul City Hospital, where practitioners from Norway, Sweden and Denmark would spend their overseas fellowship years in an effort to craft a new style of clinical facility.^35^ Collectively, this frenzy of biomedical activity lent itself to the image of a newly transformed medical community, although the underlying reality remained complex, with strong continuities from colonial rule.

Prior to the creation of KAPE (Korean Association of Parasite Eradication, 1964), the domestic effort lay with addressing endemic forms of disease, especially tuberculosis, leprosy and cholera, problems with a lengthy history.^36^ The Ministry of Health and Social Welfare devoted its limited resources to identifying and refurbishing rural clinics and other available sites, with major urban areas attracting the bulk of such materials. As with doctors, training for nurses was also at a premium, with the University of Indiana-Bloomington serving as a liaison late in the decade of the 1950s, seeking to strengthen its networks in Asia.^37^ This last point, although it may appear to lie outside the scope of South Korea, remains highly relevant; for many of the partners, whether new or of great duration, the Republic of Korea (ROK) mission lay within a context of mobilising the ‘Free World’, or anti-Communist Asia, reaching out to include Southeast and East Asian partners.^38^

## Emerging Specialists in Parasitology

Within this context, the anti-parasite programme was marketed as a domestic campaign to a South Korean audience, and it would be led by its brightest and best specialists. Dr Seo Byung-Seol (1921–91), who later presented as a Korean representative at the first APCO conference in 1974, was among the most prominent of these individuals, a leading parasitologist at the national and regional levels. Seo was a late 1940s graduate of the newly formed Seoul National University (1946–present), meaning that he began his university education under the Japanese imperial system.^39^ Following the Korean War, he travelled to the USA for a year of overseas study (1955) at the University of Minnesota, where he continued to develop his interests in parasites and ecology. Focusing on a range of worms and intestinal parasites, Seo was almost ideally positioned at the beginning of the 1960s, certainly in terms of linking his personal interests to those of KAPE. Similarly, his colleague at Yonsei University and the Vice-President of KAPE, Soh Chin Thack (1921–2016), later founded that university’s tropical medicine programme (1957) and spent a year abroad at Tulane University in the early 1960s.

When KAPE formed in the early 1960s, it reflected recent trends, even as its trajectory points to a much longer history, arguably going back to the colonial period. First, Park Chung Hee reconfigured a number of public health efforts lingering from the Syngman Rhee era (1948–60), and this strategy proved to be hugely advantageous for those with the right kinds of expertise, especially younger scholars like Seo and Soh. Secondly, although the number of those trained in parasitology remained small, there was a surrounding community of doctors and health professionals, meaning that KAPE was not composed of specialists only, and included a number of interested parties (medical doctors, biologists), often with related professional interests. At its inception, KAPE studied and targeted a number of pests, reflecting its origins in an agrarian society, where rats and pests posed a threat to the grain supply.

With this aim, KAPE joined a number of related public health concerns on the national agenda. As mentioned, these items included leprosy, cholera, tuberculosis and the building of a public health network. To this end, FP also took shape under the banner of a new national programme, although it, too, had a lengthy prior history, both for the colonial period and the Rhee years. For the early 1960s, this meant a series of trial efforts, including the Koyang study (rural) overseen by Yang Jae-Mo of Yonsei University and the Sundong-gu study (urban), run by Kwon E-Hyock of Seoul National University.^40^ Together, these two mini-studies provided a baseline for what became a national FP network by the middle of the decade, one aiming to distribute information about reproductive technologies, along with the objects themselves, especially the Lippes’ loop for women.^41^ This plastic intrauterine device (IUD) became the primary vehicle of birth control for many international FP campaigns of the period.

Still a relatively poor nation, South Korea received international support for this mission, especially from the Population Council.^42^ The same would hold true for KAPE, as it required a great deal of infrastructure to accomplish its ambitions, especially at the national level. In April 1966, the ROK passed a law calling attention to the treatment of parasitic diseases, a step in the direction of implementing new plans.^43^ Before a national plan could be attempted, however, the organisation needed the necessary hardware (microscopes, slides, a means of gathering samples) and human element (willing subjects or participants, a trained labour force to gather materials, a biosciences team to process and analyse samples) to pursue such an effort. In the wake of Korea’s normalisation with Japan in 1965, the signing of related agreements made such exchange possible, and specifically, medical exchange came to fruition with the signing of an agreement in 1968, furnishing a significant portion of the ROK network. Japan’s technical influence, initially reduced after 1945, was reintroduced with this gesture.^44^

## From Colonialism to Development: Japan–ROK Medical Cooperation Takes Shape in Cold War Asia

The legal and institutional infrastructure enabling medical exchange between Japan and South Korea was established quickly in the mid-1960s through the process of normalising diplomatic relations. As part of the Japan Republic of Korea Basic Relations Treaty in December 1965, the two countries signed the ‘Agreement on the settlement of problems concerning property and claims and on economic co-operation’ in Tokyo on 22 June 1965.^45^ The Agreement stipulated that South Korea was to receive 300 million dollars’ worth of Japanese products and service gratis and a loan of 200 million dollars in yen over the next decade. Additionally, a note accompanied with the Agreement also stated that a more than 300 million dollars civilian loan should be passed on from Japan to South Korea. Based on the Agreement, in March 1966, South Korea became the first country that received a yen credit from the Japanese Overseas Economic Cooperation Fund (OECF, established in March 1961).^46^

The Japanese economic assistance to South Korea resulted from Japan’s shifting position in East Asia during the Cold War from a colonial power to a reliable American ally.^47^ In the early 1950s, pressed by the Cold War exigencies, the USA strove to establish an interdependent ‘free world’ alliance buttressed by economic assistance and development aid.^48^ From the American perspective, Japan and South Korea were valued allies forming a critical buffer zone in the ever-more destabilised region first with the Korean, and later the Vietnam Wars. To secure the alliance, over the 1950s and 1960s, the USA offered a tremendous amount of economic assistance to Japan, and both military and economic assistance to South Korea. However, from the mid-late 1960s, amidst financial difficulties, the USA came to consider reducing military assistance to South Korea. As withdrawal from Vietnam loomed as a real possibility, the strategic importance of South Korean military capability became less urgent to the USA. Just at the time, Japan was going through an unprecedented economic boom. Under the circumstances, the USA suggested Japan to join in the US effort to offer economic assistance to South Korea. Japan welcomed the proposal from the USA. The government was cognizant of the continued importance of its former colony in the region’s geopolitics.^49^ Moreover, leaders of Japanese economy and industry strongly wished to expand the market in South Korea through economic assistance.^50^ Finally, South Korea also was not averse to the proposal. The government’s First Five-Year Economic Development Plan was coming to an end in 1966. In 1967, it was to launch the Second Five-Year Plan, and it was viewed that Japanese assistance would be useful to that end. Interests of the involved parties converged in Asia under the Cold War. Consequently, the Japanese economic assistance to South Korea began in the mid-1960s.

A call for bilateral medical exchange came against this specific backdrop.^51^ In the spring of 1968, the South Korean government requested Japan for medical cooperation in parasite eradication.^52^ Responding to the request, the OTCA dispatched a small survey group to South Korea consisting of a staff each from the OTCA, Ministry of Health and Welfare, Ministry of Foreign Affairs, as well as two parasitologists, Yokogawa Muneo and group leader Amatsuru Masamitsu. The group toured Seoul, Pusan and Daegu between 24 June and 8 July, met the experts at Yonsei and Seoul National Universities and those affiliated with KAPE and finally produced and signed the Record of Discussion with the South Korean delegate, summarising the content of medical cooperation. Following the publication of the plan of Japan’s medical assistance to South Korea’s anti-parasite campaign on 10 August 1968, members of the survey group met the South Korean delegate in Tokyo on 17 November.^53^ The meeting, also attended by Seo, decided specifically on the actual staff and goods involved in the first 2 years of cooperation.^54^

What is particularly fascinating about the bilateral medical cooperation was how communications at the grassroots level were just as constitutive of the government initiative as the official dialogue. In 1964, as the high-rank officials from the two countries were deliberating over the terms of economic cooperation, Soh invited his Japanese colleagues Hasegawa Shūji at Gunma University and Sasaki Manabu at Tokyo University to South Korea to discuss possibilities for Japanese aid being used for the South Korean anti-parasite campaign. At the meeting, the Japanese parasitologists suggested that the KAPE contact Kunii. In May 1965, KAPE approached Kunii who was in Seoul attending the IPPF Western Pacific conference on FP. Eventually, KAPE invited Kunii to visit its office and examination clinics on 27 May 1965 and asked if Japan could provide aid for the nationwide anti-parasite campaign to be launched after April 1966 with the establishment of the Parasite Diseases Prevention Law.^55^ In return, JAPPI invited KAPE President Lee Young-joon to report on the situation in South Korea surrounding measures against parasite diseases at the JAPPI’s national congress in Tokyo on 29–30 July 1965. Responding to Lee’s speech, in October 1966, the JAPPI dispatched a survey group to South Korea, which included Kunii. The report from the trip asking for the Japanese aid was submitted to various government agencies, including the OTCA and the Medical Cooperation Committee of the ruling party Liberal Democratic Party (LDP). Based on the JAPPI report, in November 1967, the member of the LDP Committee Shirahama Nikichi visited South Korea. After the lobbying of Shirahama and his colleagues in the government agencies, the Japanese government allocated a budget for medical cooperation in the South Korean anti-parasite campaign for the fiscal year of 1968. The series of transnational grassroots dialogues therefore catalysed bilateral medical cooperation.^56^

Even after the bilateral scheme began, health activists from both countries also played a pivotal role as medical experts or as indispensable partners to the medics. For instance, Kunii travelled to South Korea in 1971 as a member of the OTCA’s survey group. In turn, Seo was often present at the official meetings side-by-side with the South Korean health official accountable for the bilateral scheme.^57^ These civilian participants were so integrated into the bilateral scheme that the boundaries between the governmental or non-governmental factions were often invisible.

As part of the official anti-parasite campaign, goods and medical personnel travelled across Japan and South Korea for a decade after the mid-1960s. For instance, in the first fiscal year of 1968, the following made-in-Japan products crossed the Sea of Japan to South Korea:

6 Station Wagon cars for health check-ups (Toyota Automobiles);
120 Monocular microscopes (Olympus Optics);
6 Binocular microscopes (Olympus Optics);
60 Centrifuge machines (producer not noted);
2,000 Tablets × 250 Boxes of Anthelmintic Yuismin (Nihon Shin’an Pharmaceuticals).^58^

The donation of these goods was accompanied by the training of one doctor, four technicians and three administrators from South Korea in Japan and by the travel of two Japanese doctors to South Korea to train their colleagues for 3 months in such techniques as with Kato’s thick smear technique, which was originally introduced by Japanese Kato Kan and Miura Momoshige in 1954.^59^ These goods and personnel became an integral part of the South Korean anti-parasite campaign in the late 1960s and early 1970s.

The background to the rollout of South Korea’s school campaigns thus carries with it a great deal of historical irony. The microscopes, slides and basic technologies used for much of the anti-parasite effort came from Japan. The Korean medical technicians trained to process samples completed a 2-year degree, with much of the pedagogy deriving from Japan. These individuals, along with the technology, furnished some of the most representative images from the period, typically featuring a group of young women poised in front of a microscope, with a crowd of curious onlookers. Just as there was often resistance to public health initiatives in the colonial period, some Koreans sought to evade the scrutiny of the state, either by skipping school or by substituting the stool sample of a pet. In either case, the focus on schools captured a critical segment of the population.

If the Cold War provided a political framework for the bilateral medical cooperation, Japanese colonialism shaped the perspective and the actual terms of the cooperation as a post-war development project. The legacy of Japanese colonialism was substantiated within the post-war cooperation in three significant ways. First, medical cooperation relied on the network forged during the colonial period. Soh felt he could approach his Japanese colleagues and ask for aid precisely because both parties were in the network of parasitologists already established under Japanese colonialism.^60^ The second was the linguistic capability of South Korean participants educated under the colonial education system. Lee, for instance, was fluent in Japanese due to his training during the colonial period. Reportedly, he corrected the Japanese interpreter’s translation during his speech at the aforementioned JAPPI congress in 1965.^61^ Allegedly Lee was able to garner support among his Japanese colleagues because they were impressed with his Japanese proficiency. Lee’s language skills therefore facilitated the bilateral medical cooperation.^62^

Finally, the notion of Asian brotherhood, a style of fraternalism Japanese colonisers often used to portray the relationship between themselves and Koreans as colonial subjects, was evident in how the Japanese participants characterised the role of medical cooperation in the bilateral diplomacy. Reflecting on the cooperation, Amatsuru likened the relationship between the two countries to brothers sharing an awkward past, and with an uplifting tone characterised medical cooperation as a panacea for overcoming the potentially tense diplomatic tie that exists between the two:

[Medical cooperation] shows how we can find ways to trust each other while overcoming the awkwardness that had existed for long. As the proverb ‘Brothers will be united to defend themselves against the enemy even if they might fight otherwise’ suggests, it is in no ways normal that Japan currently distances itself from South Korea in some respects while keeping it close in others. We hope [medical cooperation in the anti-parasite campaign] will be an opportunity to establish the system of cooperation in various other medical fields.^63^

Particularly noteworthy in Amatsuru’s wording was that the two countries compared as brothers were in no ways on an equal partnership. Instead, they were on a hierarchical relationship in which Japan assumed the role of the bigger brother. While it could well be that Amatsuru wished to convey a sense of goodwill with this statement, this portrayal of hierarchical fraternity was reminiscent of the kind of argument observed in the Japanese articulation of pan-Asianism during the period of its colonial rule in, for instance, the Greater East Asia Co-Prosperity Sphere propaganda that legitimated the Japanese Empire and its wartime aggression in Asia.^64^ As described subsequently, the legacies of Japanese colonialism integrated in medical cooperation were significant precisely because they not only mobilised the cooperation itself but also helped to cement the vision of post-war regional cooperation in Asia in which Japan’s role as Asia’s benevolent donor nation was assumed in part due to its heritage as the only non-western colonial power in the region that used the idea of cultural and racial affinities with its colonial subjects.^65^

## Korean Military/Civic Medical Aid to Vietnam

Japan’s interest in linking with its Asian neighbours took place prior to, and simultaneously with, the expansion of the Vietnam War. Prior to American involvement (1965), regional cooperation provided opportunities for new actors to expand their ambitions. For South Korea, participation in the war encouraged the impulse to provide supplemental medical care for Vietnamese, whether civilian or military. In particular, civilian populations provided the focus for any number of activities, including enthusiastic vaccination campaigns, senior care and neonatal care.^66^ Young children also received a significant number of free haircuts from South Korean forces, intended as a sign of good will but equally, offered as a public health measure likely directed against typhus.

Along with these types of campaigns, South Korean civic action provided a series of building initiatives, with much of this activity coming under the auspices of the Pigeon (or ‘Peace Dove’) Brigade. These support troops built schools, training centres, temporary housing for orphans and infrastructure, such as local roads.^67^ Once the structures were complete, the new sites were used to train Vietnamese workers in a number of areas, including hand-intensive labour (sewing, textiles) and the acquisition of office skills, such as typing. With the close of the Korean War a decade earlier, American and international aid organisations had conducted similar activities throughout South Korea, especially in the recovery period (1954–60) associated with Syngman Rhee. Having experienced these forms of assistance, Korean personnel internalised the logic and now sought to take on a new role, this time as the donor. In this sense, they matched Japan’s ambition to conduct technical outreach with Asian partners.^68^

Throughout the Vietnam period, Korean workers, engineers, technicians and soldiers had to confront this emerging power dynamic, learning how to interact with other Asians in new circumstances, especially in work, medical and social situations. The types of medical and civic outreach programmes named here did not constitute ‘aid’, at least in a formal sense: the terms of the encounter were shaped by the surrounding military context. This power inequity shaped much of the labour policy during Vietnam, with thousands of TCN (third country nationals) workers arriving on short-term contracts. In the majority of these cases, American contractors assumed the lead role, with much of the ‘on the ground’ work conducted by subcontractors, typically Japanese and Korean firms. In terms of daily experience, this meant that Vietnamese labourers experienced supervision by other Asians, frequently by Japanese and Korean overseers, while working alongside large numbers of Filipino and Cambodian colleagues.^69^

For the Korean labour force specifically, a number of historians have noted that this situation was not entirely new, especially given the long history of contact between neighbours within the Sinophone world, as well as more recently, with Koreans serving in the Japanese military during the Pacific War.^70^ Still, this was the first time abroad for many of these Koreans in the role of post-colonial subject, especially for more senior personnel, many of whom had prior training and education under the Japanese system. While interacting with Vietnamese colleagues, their labour and identity as ‘South Koreans’ were very much in process, actively being renegotiated. Especially for medical encounters, the legacy of existing frames for understanding, particularly that of Japanese imperial medicine, provided a partial basis for understanding the new setting and the actors.

Within the contingent circumstances of wartime, this developmental impulse took the shape of numerous outreach programmes, as mentioned earlier, especially those directed through the Pigeon Brigade. Korean public health officials also undertook extensive inquiries about Vietnamese villages and their waste disposal methods.^71^ This activity amounted to a comprehensive description of Vietnamese toilet behaviour, and while it held obvious uses for public health applications, the information resembled a comprehensive ethnography, a self-justification on the part of Korean officials. During the late stages of the war, Korean healthcare professionals received a contract from USAID to conduct their activities under the label, KOPREM or ‘Korean preventive medicine’.^72^ Specifically, KOPREM ran during the early 1970s and placed officials in Vietnamese villages to track figures for disease incidence, watching for outbreaks of contagious disease.^73^ In a sense, the American military placed the Koreans in this unusual position as a mediating or sub-imperial body, serving as an on-the-ground presence to monitor for problems.^74^ In fact, American officials at the time explained this role precisely in terms of Korean experience with their own villages over the previous decade (1955–65), arguing that such a background would translate well to the Vietnamese setting.^75^

With this wartime experience immediately preceding APCO, formed in 1974, it should not be surprising that were numerous continuities. In the domestic sphere, many of the medical campaigns reaching South Korean villages in the *Saemaul Undong* (New Village Movement) meant that there was an identification between rural Koreans and comparable communities in Vietnam, at least from the perspective of Korean elites. These villages were targeted to receive better housing, FP and public health initiatives, with the government seeking to provide a means of self-empowerment. APCO, too, held certain parallels with the Vietnam experience. In effect, the Koreans sought to repackage their own developmental trajectory, along with the more recent wartime experience, and to pass it along to Southeast Asian partners. That is, they had learned from the post-Korean War experience and wanted to use this as a basis for their own form of outreach. Seo, for example, viewed Vietnam as a chance to study the movement of parasites, representing an experimental context in which to refine his findings.

## When Parasite Control Met FP: The Asian Parasite Control Organisation and Integration Project

The dialogues between Japan and South Korea under bilateral medical cooperation ultimately gave birth to the APCO, established in October 1974 at its inaugural meeting in Tokyo.^76^

APCO was a non-governmental organisation founded by Kunii and his fellow activists and medical experts involved in parasite eradication in Japan, Korea and Taiwan, including Seo.^77^ In addition to parasite control, APCO had another grand mission that was less obvious from the organisation’s title: to integrate FP with parasite control. For this reason, the APCO invited L. S. Sody, the Secretary of Inter-Governmental Coordinating Committee Southeast Asia Regional Cooperation in Family and Population Planning (IGCC) to the inaugural meeting as an honourable guest.^78^ By the second meeting, APCO clearly articulated this mission as its ultimate goal. Experts, officials and activists discussed how to introduce FP as part of the public health project to eradicate and prevent diseases caused by intestinal parasites. FP was as represented as parasite control in APCO, contrary to what the organisation’s name indicated.

APCO’s rather unique perspective originated from Kunii. Kunii in the mid-1960s led two separate lives mentioned earlier, as a health activist dedicated to parasite control and as a FP activist. Over the 1960s, changing attitudes within the Japanese government to Official Development Assistance (ODA) exhorted Kunii to consider combining his hitherto disparate causes into one ambitious development initiative. The first was the government’s increased involvement in parasite eradication as ODA. In 1970, based on the experience in South Korea, the OTCA launched a similar parasite control initiative in Taiwan’s Nantou County. The initiative catalysed not only dialogue between the Japanese and Taiwanese involved in the initiative but also a three-way conversation among the Japanese, Taiwanese and Koreans who were still carrying out its/their own initiative.^79^ This three-way communication paved a way for Kunii to develop his own network, which ultimately helped him to establish APCO.

The second was the Japanese government’s decision to participate in international cooperation in FP. In the mid-1960s, FP spread globally as a subject in ODA, especially after the USAID began to subscribe to Rostovian modernisation theory and invest in its FP initiatives in developing countries to catalyse socioeconomic development.^80^ The Japanese government officially joined in the cause when Prime Minister Eisaku Sato responded to John D. Rockefeller’s call to sign the Declaration on Population, which was presented to United Nations Secretary-General U Thant on 10 December 1966.^81^ The Japanese move was fostered even further when American William H. Draper, a member of the Governing Body of the IPPF, visited Japan in the summer of 1968.^82^ In Japan, Draper tirelessly met the country’s political leaders, including Sato and former Prime Minister Kishi Nobusuke, to garner support for international cooperation in FP, especially for Asia. After Draper’s visit, the government decided to donate 100,000 dollars annually to the IPPF from the fiscal year of 1969 and at the same time, let the OTCA launch FP projects in Southeast Asia, notably, Indonesia, the Philippines, Thailand and Bangladesh.^83^ Draper’s Japan tour also persuaded Kishi and successful businessman and philanthropist Sasakawa Ryōsuke to assist the cause, which eventually led to the Japanese government’s approval for the establishment of JOICFP. The tide within the government broadened Kunii’s horizon to consider developing his activism as relevant to ODA.^84^

Against this backdrop, Kunii thought of merging parasite control with FP under a single development initiative. Kunii recalled he had conceived of the idea in February 1973 when he observed in a village in the Philippines that nutrition advice was given there as an incentive for FP.^85^ Kunii was impressed with the initiative precisely because it tackled the problem of high infant mortality and morbidity rates simultaneously with reducing birth rates. Upon returning home, Kunii devised his own FP initiative by applying the Philippines method and by basing it on his expertise in parasite control. He called it Integration Project (IP).

Kunii presented three reasons to justify the integration of parasite control and FP. First, infections of intestinal parasites were endemic in the area often targeted by FP initiatives, so odds were that the majority of people whom the FP initiatives wished to reach would have children suffering from the infection. Secondly, anti-parasitic drugs often generated dramatic, tangible and quick results, so would work as a perfect tool with which to show people their goodwill. Kunii also claimed that health workers could forge rapport with people through a parasite eradication programme, which could further facilitate the process to introduce FP. Thirdly, parasite control could be conducted fairly at a low cost as it required relatively little equipment. With these reasons, Kunii tried to legitimate the IP.^86^

After his Philippines tour, Kunii quickly laid the groundwork to materialise his idea into practice. He approached the existing contacts in South Korea about the possibility of forming a new pan-Asian organisation dedicated to parasite control, and consequently, Seo and Lee Young-joon agreed to be the founding members of the APCO.^87^ Furthermore, in July 1974, using the connection with JICA, Kunii visited Taipei and met Wang Jinmao (Minister of Health), Hu Huide (Director of Department of Health, Taiwan Provincial Government) and Zeng Baicun (Director of the Institute of Malaria, Taiwan Provincial Government). He suggested them to transform the area in Nantou where JICA was conducting its parasite eradication initiative into a model district for the IP.^88^ The negotiation was successful, thus JOICFP launched the first pilot project in Nantou in July 1975, which was funded by Sasakawa’s Japan Shipbuilding Industry Foundation.

The Nantou project was quickly followed by other pilot projects in other countries of the APCO members, with financial support from the JOICFP and the IPPF. Of those, the pilot project in Indonesia was particularly significant, as it was the first IP pilot project organised in Southeast Asia that was strongly shaped by Japanese interests. Indonesia was the first country in Southeast Asia where JICA deployed project-based medical cooperation in FP (from 1969), in part because the Japanese government regarded development aid/economic assistance as an effective means with which to resolve the issues of war reparation with Indonesia.^89^ For this reason, by the time APCO approached the country on the possibility of organising a pilot project in Indonesia, there was a network that would facilitate a Japan-oriented development project there. Based on the infrastructure laid by the JICA, APCO organised the IP pilot project from 1976, first in the mining town of Sawah Lunto, which later expanded to reach out to the greater District of Sawah Lunto-Sijunjung.

The Sawah Lunto project was not only unidirectionally imposed by APCO but it was a marriage of convenience between the APCO and the Indonesian government and health campaigners who also saw the health project’s benefits, albeit from distinctive perspectives. With regard to FP, Indonesia had experienced a civic birth control movement already in 1953, which led to the establishment of the Indonesian Planned Parenthood Association in 1957. However, the effect of the movement during the period was limited, in part because of the unfavourable political environment. With the political change in 1965, the government became more receptive to the idea of national FP programmes, symbolised by the signing of the aforementioned ‘Declaration on Population’ by Suharto in 1967. Based on the event, in December 1968, the half-private, half-governmental National Family Institute was established, which was replaced by the governmental National Family Planning Coordinating Board, NFPCB, in 1970. In parallel, non-governmental health and religious organisations, including the Indonesia Christian Church, Association of Voluntary Health Services of Indonesia (Persatuan Karya Dharma Kesehatan Indonesia Perdhaki) and Indonesia Islamic Reform Organisation (Muhammadiyah) widely collaborated with the government on the cause.^90^ Therefore, when APCO considered deploying a pilot project in Indonesia, the country had already been familiar with a FP initiative.

Similar to the South Korean project in Vietnam, the IP pilot project in Indonesia—and more generally the IP projects in Southeast Asia—were constructed through the notion of shared traditions. For instance, assessing the Indonesian IP pilot project, Kunii specifically mentioned the traditional custom of mutual aid, *gotong-royong*, as an important element within the project. Kunii then compared *gotong-royong* to the traditional social organisation in Japan that became the basis for the Agricultural Cooperative, the biggest agricultural association in Japan, as well as to the community organisation supporting the *Saemaul Undong in South Korea*. Finally, he concluded, ‘these organizations [exhorting] solidarity among the residents are a character of rural community which is very Asian’, and ‘defending, expanding and encouraging such a community organization is key to the success of the Integration Program [*sic*]’.^91^ This portrayal of social practices in Southeast Asia with the emphasis on shared Asian identity was central to Kunii's characterisation of the IP.

On the individual level, this portrayal reflected Kunii’s commitment to the grassroots, community-based health project, which in part was shaped by his humanism. Elsewhere where there was an opportunity, Kunii repeated that everyday people and local communities—not the nation-state or global politics—must be at the centre of any development project.^92^ On the other, the perspective to pursue shared heritage in what in reality were quite diverse social organisations in Asia could easily coalesce with the discourse of Asian brotherhood, which, as mentioned earlier, buttressed the post-war ROK-Japanese medical cooperation, as well as the Japanese articulation of Pan-Asianism during the period of colonial rule. APCO, therefore, was undergirded by convoluted narratives of Asianism influenced both by the burgeoning post-war ideology, as well as the legacy of the Japanese colonialism.

## Conclusion

The threads connecting pre-war and wartime networks of parasitology and tropical medicine to post-war medical aid and developmentalism helped to shape the aid practice of both Japan and South Korea. As we have noted, these networks were frequently informal, and certainly we do not want to argue the case of a teleology or an inevitable outcome. Rather, the confluence of these networks of colleagues brought together Japanese parasitology and a subset of their Korean colleagues, many of whom had prior connections within the imperial university system. The Southeast Asian context next provided an opportunity to explore and share the developmental expertise acquired, with the resulting IP fusing Kunii’s parasitology and FP in an unusual hybrid package. For the Koreans, very recently in Vietnam, the chance to participate in APCO continued the process of post-Korean War recovery, as well as an additional chance to explore and experiment with forms of practice to be used in the domestic context.

A recent dissertation by Young Su Park takes up the question of contemporary Korean health ambitions in Ethiopia, arguing that, despite good intentions, there is often a lack of fit between the East African context and the aims of such developmentalism.^93^ In contrast, the APCO project did not raise such concerns in 1974, as the two major actors, Japan and South Korea, assumed that their developmental experience could easily transfer.^94^ Moreover, geographical and temporal proximity, along with prior relationships established under circumstances of colonial and wartime rule (Taiwan, Indonesia), made for a relationship of collegiality, one endorsed with the language of a post-war Pan-Asianism. For Japan’s JOICFP and JICA, and Korea’s own formative institutions (KAPE, and later, KOICA), still in process, the question remains concerning the extent to which this brand of pan-Asianism carried with it powerful, uncomfortable echoes from an earlier time.

## Footnotes

1 Uma Kothari, ed., *A Radical History of Development Studies: Individuals, Institutions and Ideologies* (London: Zed, 2005).

2 Karl Ittmann, *A Problem of Great Importance: Population, Race, and Power in the British Empire, 1918–1973* (Berkeley and London: University of California Press, 2013).

3 Liat Kozma, ‘Between Colonial, National, and International Medicine: The Case of *Bejel*’, *Bulletin of the History of Medicine*, 2017, 91, 744–71.

4 Hiromi Mizuno, Aaron S. Moore and John DiMoia, eds, *Engineering Asia: Technology, Colonial Development, and the Cold War Order* (London: Bloomsbury Academic, 2018); Aaron Stephen Moore, ‘Japanese Development Consultancies and Postcolonial Power in Southeast Asia: The Case of Burma’s Balu Chaung Hydropower Project’, *East Asian Science, Technology and Society*, 2014, 8, 297–322; Aaron Stephen Moore, *Constructing East Asia: Technology, Ideology, and Empire in Japan’s Wartime Era, 1931–1945* (Stanford: Stanford University Press, 2013); Aya Homei, ‘Between the West and Asia: “Humanistic” Japanese Family Planning in the Cold War’, *East Asian Science, Technology and Society*, 2016, 10, 445–67; J. Megan Greene, *The Origins of the Developmental State in Taiwan: Science Policy and the Quest for Modernization* (Cambridge, MA: Harvard University Press, 2008).

5 Mizuno, Moore and DiMoia, *Engineering Asia*, pp. 1–42. .

6 Some scholars use the term ‘re-building’ to emphasise the legacy of Chosòn\Korea, but we have opted to use ‘nation-building’ specifically to emphasise post-colonial South Korea.

7 John P. DiMoia, *Reconstructing Bodies Biomedicine, Health, and Nation-Building in South Korea since 1945* (Stanford: Stanford University Press, 2013); Christopher Aldous and Akihito Suzuki, *Reforming Public Health in Occupied Japan, 1945–52: Alien Prescriptions?* (Abingdon and New York: Routledge, 2012).

8 For our understanding of colonial medicine, works such as Michael Shiyung Liu’s that stress networks for the formulation of medical practices under the Japanese rule have been very useful. While we acknowledge the significance of focusing on ideologies, as well as issues of violence, resistance and cooperation in order to clarify the ways in which medicine was performed vis-à-vis Japanese colonialism, the limited scope of this article has prevented us from drawing a comprehensive picture of medicine under the colonial Japanese rule. For the reflection of colonial medicine, see, for example, Michael Shiyung Liu, *Prescribing Colonization: The Role of Medical Practices and Policies in Japan-Ruled Taiwan, 1895–1945* (Ann Arbor, MI: Association for Asian Studies, 2009); Ming-cheng Miriam Lo, *Doctors Within Borders: Profession, Ethnicity, and Modernity in Colonial Taiwan* (Berkeley: University of California Press, 2002).

9 Barak Kushner and Sherzod Muminov, eds, *The Dismantling of Japan’s Empire in East Asia: Deimperialization, Postwar Legitimation and Imperial Afterlife* (London: Routledge, 2017).

10 Howard Chiang, ‘From Postcolonial to Subimperial Formations of Medicine: Superregional Perspectives from Taiwan and Korea’, *East Asian Science, Technology and Society*, 2017, 11, 469–75.

11 Junho Jung and Kim Ock-joo ‘“It All Started from Worms”: Korea-Japan Parasite Control Cooperation and Asian Network, 1960s – 1980s’, *Uisahak*, 2018, 27, 49–88. See also Park Young-Jin, ‘Han’guk kwa Ilbon ŭi kisaengch'ung chirhwan t'oech'i ŭi yŏksa [History of the Fight against Parasites, Korea and Japan]’ (MA Thesis, Seoul National University, 2016).

12 Academic works on public health since the Meiji period abound, but the most concise is among the English language literature Aldous and Suzuki, *Reforming Public Health in Occupied Japan, 1945–52*.

13 For the cultural representation of public health and disciplining of the bodies in post-war Japan, see, for example, Yoshikuni Igarashi, *Bodies of Memory: Narrative of War in Postwar Japanese Culture* (Princeton: Princeton University Press, 2000).

14 Aldous and Suzuki, *Reforming Public Health in Occupied Japan, 1945–52*.

15 For the state of parasite infections during this period, see Noboru Kagei and Shigeo Hayashi, ‘Control of Parasites in Japan’, in M. Otsuru, S. Kamegai and S. Hayashi, eds, *Progress of Medical Parasitology in Japan* (Tokyo: Meguro Parasitological Museum, 2003), VIII, 649–69.

16 Among the parasite infections, Japanese encephalitis B and malaria were also high on priority among the government. For the English-language work on public health in post-war Japan, see, for example, Aldous and Suzuki, *Reforming Public Health in Occupied Japan, 1945–52* ; Seiji Tanaka *et al.*, ‘Acute Infectious Diseases in Occupied Japan’, *Nihon ishigaku zasshi*, 2007, 53, 229–48.

17 This movement was assisted by various Japanese and non-Japanese sympathisers and specialists of China, and this included the American journalist Edgar Snow, who had been reporting on Chinese politics since the 1920s. John Maxwell Hamilton, *Edgar Snow, A Biography* (Bloomington: Indiana University Press, 1988).

18 Chōjirō Kunii, *Watashi no sengo undōshi: Roman to genjitsu no hazama de [History of My Post-war Activism: Between Romanticism and Reality]* (Tokyo: Hoken kaikan, 1977).

19 Since there was not yet an established screen method or a patented medicine against the intestinal parasite infection, the campaign at the beginning was experimental, to say the least. Kunii later recalled he and other campaigners used clamshells to collect stool samples and generic medicine containing tetrachloroethylene to eradicate the infection. Wataru kunii vol. 1, 287–91, hara no mushi sentoki, pp. 8–12.

20 For the reform of public health centres, see Sey Nishimura, 'Promoting Health During the American Occupation of Japan The Public Health Section, Kyoto Military Government Team, 1945–1949', *American Journal of Public Health*, 2008, 98, 424–34.

21 This is not to suggest that the state suddenly endorsed an open-arm policy on birth control. To the contrary, the state continued to hold an ambiguous view towards the birth control movement as in the case of the pre-war period and wartime, which was epitomised by the incessant policy debate over the validity of adopting birth control to regulate population dynamics occurring throughout the late 1940s and early 1950s. For more, see, for example, Aiko Takeuchi-Demirci, *Contraceptive Diplomacy: Reproductive Politics and Imperial Ambitions in the United States and America* (Stanford: Stanford University Press, 2018); Yasuko Tama, ‘Jutai chōsetsu (bāsu kontorōru) to botai hogohō [Birth Control and the Laws to Protect Mothers’ Bodies]', in Chiaki Shirai, ed., *Umi sodate to josan no rekishi - kindaika no 200-nen wo furikaeru* [*History of Birth, Childcare and Midwifery: Looking Back 200 Years of Modernization*] (Tokyo: Igaku shoin, 2016), 110–35; Miho Ogino, *‘Kazoku keikaku’ eno michi: Kindai nihon no seishoku wo meguru seiji* [*Paths to ‘Family Planning’: Politics around Reproduction in Modern Japan*] (Tokyo: Iwanami Shoten, 2008) ; Yasuko Tama, *‘Kindai kazoku’ to bodī poritikkusu* [*‘Modern Family’ and Body Politics*] (Kyoto: Sekai shisōsha, 2006); Michiko Obayashi, ‘Sengo nihon no kazoku keikaku fukyū katei nikansuru kenkyū [A Study on the Popularization of Family Planning in the Post-War Period]’ (unpublished PhD thesis, Ochanomizu University, 2006); Christiana A. E. Norgren, *Abortion before Birth Control: The Politics of Reproduction in Postwar Japan* (Princeton: Princeton University Press, 2001).

22 Sujin Lee, ‘Problematizing Population: Politics of Birth Control and Eugenics in Interwar Japan’ (PhD thesis, Cornell University, 2017); Aiko Takeuchi-Demirci, ‘Birth Control and Socialism: The Frustration of Margaret Sanger and Ishimoto Shizue’s Mission’, *Journal of American-East Asian Relations*, 2010, 17, 257–80; Karen Lee Callahan, ‘Dangerous Devices, Mysterious Times: Men, Women, and Birth Control in Early Twentieth-Century Japan’ (PhD thesis, University of California, Berkeley, 2004); Elise K. Tipton, ‘Birth Control and the Population Problem’, in Elise K. Tipton, ed., *Society and the State in Interwar Japan* (London and New York: Routledge, 1997), 42–62; Elise K. Tipton, ‘Birth Control and the Population Problem in Prewar and Wartime Japan’, *Japanese Studies*, 1994, 14, 54–64.

23 Obayashi, ‘Sengo nihon’, 163.

24 The Japanese name of the organisation was Nihon Kazoku Keikaku Fukyū Kyōkai.

25 Obayashi, ‘Sengo nihon', 174.

26 Some members held an ambiguous view to the business, considering that the cause, based on high morality, might degenerate into a money-mongering commercial endeavour. Also, the criticism came from powerful pharmaceutical companies that also sold condoms that Kunii’s activism prevented them from expanding their business. Obayashi, ‘Sengo nihon', 174–78.

27 Obayashi, ‘Sengo nihon', 173.

28 Sonja Kim, *Imperatives of Care: Women and Medicine in Colonial Korea* (Honolulu: University of Hawaii Press, 2019).

29 Moon Manyong and Kim Yong-Sik, *Han'guk kŭndae kwahak hyŏngsŏng kwajŏng charyo [(A) Documentary history of the development of modern science in Korea]* (Seoul: National University Press, 2004).

30 Sung-Tae Hong *et al*., ‘A Successful Experience of Soil-Transmitted Helminth Control in the Republic of Korea’, *The Korean Journal of Parasitology*, 2006, 44, 177–85.

31 John DiMoia, ‘“The Nearest Faraway Place”: South Korean Medical Out-reach and Southeast Asia as Its Research Context, 1954–1973’, *Korean Journal of the History of Sciences*, 2016, 38, 303–19. These Koreans not only developed their own medical culture, of course, but also continued to cite their Japanese professors, especially Harujiro Kobayashi, who taught parasitology at Keijio Imperial University.

32 Hong *et al*., ‘A Successful Experience of Soil-Transmitted Helminth Control in the Republic of Korea’, pp. 177–85.

33 Junho Jung and Kim Ock-joo of Seoul National University Hospital (SNUH) have published two articles on this subject as part of their ongoing research. Jung and Kim, ‘“It All Started from Worms”: Korea-Japan Parasite Control Cooperation and Asian Network, 1960s – 1980s’, pp. 49–88.

34 Kim Ock-Joo and Hwang Sang-Ik, ‘The Minnesota Project: The Influence of American Medicine on the Development of Medical Education and Medical Research in Post-War Korea’, *Korean Journal of the History of Medicine*, 2006, 9, 112–23.

35
*The National Medical Center in Korea: A Scandinavian Contribution to Medical Training and Health Development*, *1958–1968* (Oslo: Universitetsforlaget, 1971).

36 Kyuri Kim and Buhm-Soon Park, ‘Infrastructure-building for Public Health: The World Health Organization and Tuberculosis Control in South Korea, 1945–1963’, *Korean Journal of Medical History* 2019, 28, 89–138.

37 The Indiana nursing programme would run through the early 1960s. *Korea Project-ICA (International Cooperation Administration), 1959–1961*, Box 21, Indiana University, School of Nursing Records, 1914, 2002, Indiana University-Purdue University, Indianapolis (IUPUI) campus.

38
*P'yo˘nghwa wa ko˘nso˘l* [Peace and Construction] *Chuwo˘l Hankukkun Saryongbu* (Saigon, 1966).

39 Thanks to Sung-Tae Hong of the School of Public Health, Seoul National University for providing access to Dr Seo’s personal materials. For Seo’s personal story, see Chapter Five, ‘Taking Samples for the Nation’, in DiMoia’s *Reconstructing Bodies,* pp. 145–76*.*

40 As with anti-parasite activity, Seoul National University and Yonsei University, two of Korea’s most prominent institutions, tended to dominate the research activity for FP.

41 The Lippes’s loop was supplied to Korea by the Population Council. In effect, the use of the loop in international contexts provided a field test of the devise. Chikako Takeshita, *The Global Biopolitics of the IUD: How Science Constructs Contraceptive Users and Womens’ Bodies* (Cambridge, MA: MIT Press, 2011).

42 Matthew Connelly, *Fatal Misconception: The Struggle to Control World Population* (Cambridge, MA: Harvard University Press, 2008).

43 See Jung and Kim, ‘“It All Started from Worms”’ , pp. 49–88, for the details of the domestic story.

44 Jaehwan Hyun, ‘Making Postcolonial Connections: The Role of a Japanese Research Network in the Emergence of Human Genetics in South Korea, 1941–1968,’ *Korean Journal of the History of Science*, 2017, 39, 293–324. See also Hyun’s recent dissertation, ‘Yujŏnjŏk minjok mandŭlgi : Han'guk ŭi illyu yujŏn yŏn'gu, minjok chŏngch'esŏng, kŭrigo ch'ogukchŏk kwahak kyoryu 1926–2009 [The Making of a Genetic Nation: Human Heredity, National Identity, and Transnational Scientific Exchange in South Korea, 1926–2009]’ (unpublished PhD thesis, Seoul National University, 2018).

45 Victor D. Cha, *Alignment Despite Antagonism: The United States-Korea-Japan Security Triangle* (New York: Columbia University Press, 2000).

46 Hiroshi Oikawa, ‘Kankoku taigai enjo no hensen: Reshipiento kara jonā e [Changes in South Korean Overseas Aids: From Recipient to Donor],’ *Kankoku to Kitachōsen No Keizai to Seiji*, March 2016, 45–67, http://hdl.handle.net/10112/10086, accessed 29 January 2020.

47 Jin Satō and Yasutami Shimomura, eds, *The Rise of Asian Donors: Japan’s Impact on the Evolution of Emerging Donors* (Abingdon and New York: Routledge, 2012).

48 Free world network also included Thailand, South Vietnam, Taiwan and Hong Kong among others.

49 Manyong Moon, ‘Postcolonial Desire and the Tripartite Alliance in East Asia: The Hybrid Origins of a Modern Scientific and Technological System in South Korea’, in Mizuno, Moore and DiMoia, eds, *Engineering Asia*, 165–88.

50 JICA, ‘Taikan mushō shikin kyōryoku oyobi gijutsu kyōryoku [Untied Financial and Technical Cooperation for South Korea]’ (Tokyo, April 2013), 7, http://open_jicareport.jica.go.jp/pdf/1000010541.pdf, accessed 29 January 2020.

51 This also applied to other scientific fields. See Tae-Gyun Park, ‘The Role of the United States and Japan in the Development of South Korea’s Science’, *Korea Journal*, 2012, 52, 206–31.

52 Cancer research was another topic included in this round of medical cooperation.

53 JICA, ‘Taikan mushō shikin kyōryoku oyobi gijutsu kyōryoku [Untied Financial and Technical Cooperation for South Korea]’ (Tokyo, April 2013), 7, http://open_jicareport.jica.go.jp/pdf/1000010541.pdf.

54 There were three South Korean delegates altogether.

55 Wataru Kunii, *Hoken kaikan monogatari, gekan* [*Story of the Health Centre, Vol. 2*] (Tokyo: Hoken kaikan, 2001), 93–100.

56
*Ibid*.

57 Kaigai Gijutsu Kyōryoku Jigyōdan [Overseas Technical Cooperation Agency], ‘Kankoku kiseichū taisaku iryō kyōryoku jisshi chōsadan hōkokusho [Report of the Investigative Team for the Implementation of the Medical Cooperation on the Anti-Parasite Measures in South Korea]’ (Tokyo: OTCA, 1969).

58
*Ibid*., 3.

59 The thick smear method involved the use of a cellophane cover for the examination of helminth eggs in stool. Kan Kato and Momoshige Miura, ‘On the Comparison of Some Stool Examination Methods’, *Nihon kiseichūgaku zasshi [Japanese Journal of Parasitology]*, 1954, 3, 35; K. Kato, *Introduction of a Thick Smear Technique with a Cellophane Cover for Helminth Eggs in Feces* (Nagoya: Nagoya Public Medical Science Lab, 1968).

60 Kunii, *Hoken kaikan monogatari*, 63–64; Wataru Iijima, ‘The Establishment of Japanese Colonial Medicine: Infectious and Parasitic Disease Studies in Taiwan, Manchuria, and Korea under the Japanese Rule before WWII’, *Aoyama Shigaku* [Historical Studies of Aoyama Gakuin University], 2010, 28, 77–106; Wataru Iijima, ’Komento', *Kagakushi Kenkyu* [Studies on the History of Science], 2009, 48, 52–53.

61 Kunii, *Hoken kaikan monogatari, gekan*, 94.

62
*Ibid*.

63 Kaigai Gijutsu Kyōryoku Jigyōdan, ‘Kankoku kiseichū taisaku’, 12.

64 Sven Saaler and J. Victor Koschmann, *Pan-Asianism in Modern Japanese History: Colonialism, Regionalism and Borders* (London: Routledge, 2007); Dick Stegewerns, ed., *Nationalism and Internationalism in Imperial Japan: Autonomy, Asian Brotherhood, or World Citizenship* (London: Routledge Curzon, 2003).

65 Christopher P. Hanscom and Dennis Washburn, eds, *The Affect of Difference: Representations of Race in East Asian Empire* (Honolulu: University of Hawaii Press, 2016). Also, see Chiara Chiapponi, ‘Japan and the Asia-Pacific in the 1970s: From an Economic to a “Heart-to-Heart Relationship”’, *Modern Asian Studies*, 2016, 50, 1679–704; Tessa Morris-Suzuki, ‘Invisible Countries: Japan and the Asian Dream’, *Asian Studies Review* 1998, 22, 5–22.

66 DiMoia, ‘“The Nearest Faraway Place”’, 314. See also Taemin hwaltong [Civic Actions] (Saigon (Chuwo˘l Han’gukkun Saryo˘ngbu), 196? [undated])

67 The Pigeon Brigade was dedicated specifically to provide civic outreach: Taehan News, #511, Pigeon Brigade News: https://www.youtube.com/watch?v=Sf7qg26cR5g, accessed 15 December 2018.

68 Chuwo˘l Han’gukkun Saryo˘ngbu Peace and Construction. The volume offers a series of propaganda cartoons in its final pages: 48–50.

69 A. H. Lahlum, *Diary of a Contract: NBy 44105, Jan. 1962–Jun. 1967* (Saigon: RMK-BRJ, 1967). This account provides an in-depth look at the activities of American contractors in Southeast Asia, especially their labour and recruiting practices.

70 Palmer Brandon, *Fighting for the Enemy: Koreans in Japan’s War, 1937–1945* (Washington: University of Washington Press, 2013); Paul H. Kratoska, *Asian Labor in the Wartime Japanese Empire: Unknown Histories, Routledge, 2005* (London: Routledge, 2005).

71 This interest aided Seo’s studies on malaria and parasites in the Vietnam context. Byung Seol Seo, Soon Hyung Lee, Jong June Yoon and Yong Suk Ryang, ‘Parasitological Studies of Korean Forces in South Vietnam: I. Examination of Blood Films On Malaria Patients’, *The Korean Journal of Parasitology*, April 1970, 8, 25–29.

72 Mark Harrison and Sung Vin Yim, ‘War on Two Fronts: the Fight Against Parasites in Korea and Vietnam’, *Medical History*, 2017, 61, 401–23.

73 Jung-Kyun Chu, ‘Tropical Medicine in South Vietnam’, *Yonsei Reports on Tropical Medicine*, 1971, 2, 8–9.

74 Chiang, ‘From Postcolonial to Subimperial Formations of Medicine’, pp. 469–75.

75 This trope of the village was based on the work of the American-Korean Foundation.

76 Nihon Kiseichū Yobōkai APCO Kaigi Jimukyoku, *Ajia kiseichū yobō kikō (APCO) dai 1 kai kaigi hōkokusho* [Report on the First APCO Meeting] (Tokyo: Nihon Kiseichū Yobōkai, 1974).

77 For Taiwan’s involvement in the APCO, see, for example, Homei, ‘Between the West and Asia’, pp. 455–7.

78 About the IGCC, see ‘Tōnan ajia kazoku jinkō keikaku seifukan iin IGCC’, *Sekai to jinkō*, 1 July 1974, 24, 14–15.

79 Kunii, *Hoken kaikan monogatari*, 100–01.

80 Denise M. Horn, ‘Locating Security in the Womb: US Foreign Policy, Population Control and International Family Planning Programs’, *International Feminist Journal of Politics*, 2013, 15, 195–212; Alison Bashford, *Global Population: History, Geopolitics, and Life on Earth* (New York: Columbia University Press, 2014); Alison Bashford, ‘Population, Geopolitics, and International Organizations in the Mid Twentieth Century’, *Journal of World History*, 2008, 19, 327–47; John P. DiMoia, ‘“Counting One’s Allies”: The Mobilization of Demography, Population, and Family Planning in East Asia, Late 1920s–Present”’, *East Asian Science, Technology and Society: An International Journal* 2016, 10, 355–376.

81 ‘Declaration on Population: The World Leaders Statement’, *Studies in Family Planning*, January 1968, 26, 1–3.

82 ‘Kokusai undō ni kyōryoku wo! [Cooperate for the International Movement]’, *Sekai to jinkō*, 1968, 1, 12–13. On Draper, see Connelly, *Fatal Misconception*, 186–88; Peter J. Donaldson, *Nature Against Us: The United States and the World Population Crisis, 1965–1980* (Chapel Hill: University of North Carolina Press, 1990), 23–26.

83 Japan International Cooperation Agency, ‘Kaigai iryō kyōryoku jigyō jisshi keika oyobi jisseki [The Result and Progress in Overseas Medical Development Initiatives]’ (Japan International Cooperation Agency, 1975).

84 Here it must be categorically emphasised Kunii was not a passive agent. On the contrary, he was highly instrumental in persuading the government to adopt parasite control and family planning in its efforts to ODA. Kunii, *Hoken kaikan monogatari, gekan*, 60–116.

85 Chōjirō Kunii, ‘Kiseichū kujoyaku wo motsu shidōin [Instructors Who Have Medicine To Eradicate Parasites]’, *Sekai to jinkō*, 1974, 25 39.

86 Chōjirō Kunii, ‘“Kazoku keikaku - eiyō - kiseichū yobō” integurēshon no riron to jissen - moderu chiku no susumekata [Theory and Practice of the Integration of “Family Planning/Nutrition/Parasite Prevention”]’, c 1976, Wako-shi, Saitama Prefecture, Japan, Archives of the Japanese Organization for International Cooperation in Family Planning, the National Institute of Public Health, Japan [thereafter JOICFP Archives].

87 Nihon Kiseichū Yobōkai APCO Kaigi Jimukyoku, ‘Ajia kiseichū yobō kikō (APCO) dai 1 kai’, 25.

88 Kunii, *Hoken kaikan monogatari, gekan*, 127–28.

89 Hiromi Mizuno, ‘Introduction: A Kula Ring for the Flying Geese: Japan’s Technology Aid and Postwar Asia’, in Mizuno, Moore and DiMoia, eds, *Engineering Asia*, 1–40.

90 Nihon Kiseichū Yobōkai APCO Kaigi Jimukyoku, ‘Ajia kiseichū yobō kikō (APCO) dai 2 kai kaigi hōkokusho [Report on the Second APCO Meeting]’ (Tokyo: Nihon Kiseichū Yobōkai, 20 January 1976), 39.

91 Chōjirō Kunii, ‘Ningenteki kazoku keikaku no yoake [Dawn of the Humanistic Family Planning]’, October 1978, 28–29, JOICFP Archives.

92 It seems the philosophy was widely shared by other development practitioners. See, for example, Daniel Immerwahr, *Thinking Small: The United States and the Lure of Community Development* (Cambridge, MA: Harvard University Press, 2015).

93 Young Su Park, ‘TEMPOETHICS: Envisioning Asian Modernities and Enacting Global Health Projects in Ethiopia’ (unpublished PhD thesis, Stanford University, 2018).

94 Jon Sangkhamanee, *‘Rendering Ethical?: Global Saemaul Undong and the Spirit of Korean Developmentalism*’, Chulalongkorn University, talk presented at Sogang University, 28 September 2018.

## Notes

She is a co-author of *Fungal Disease in Britain and the United States 1850–2000* (with Professor Michael Worboys). Currently, Aya is working on a book manuscript titled Science for Governing* Japan’s Population*, which examines the co-productive relationship between population policies and population medicine/science.

He is the author of *Reconstructing Bodies: Biomedicine, Health, and Nation-Building since 1945* (Stanford University Press / WEAI, 2013) and along with Hiromi Mizuno and Aaron S Moore, a co-editor of the recent volume, *Engineering Asia: Technology, Colonial Development, and the Cold War Order* (Bloomsbury / WEAI, 2018).

